# Clinical and Therapeutic Predictors of Keloid Recurrence: Outcomes in a European Cohort of 206 Patients

**DOI:** 10.3390/jcm15062150

**Published:** 2026-03-11

**Authors:** Vera Vorstandlechner, Katharina Neid, Alexandra Fochtmann-Frana

**Affiliations:** Department of Plastic, Reconstructive and Aesthetic Surgery, Medical University of Vienna, 1090 Vienna, Austria

**Keywords:** keloids, scars, retrospective, recurrences

## Abstract

**Background/Objectives:** Keloids are fibroproliferative scars with high postsurgical recurrence rates and limited high-quality data from European populations. Current treatment guidelines recommend multimodal management; however, real-world practice often varies, and therapeutic efficacy in Western cohorts remains insufficiently characterized. This study aimed to analyze determinants of keloid recurrence and evaluate the impact of postoperative treatments within one of the largest Middle-European keloid cohorts to date. **Methods:** In this retrospective single-center study, 206 patients treated for at least one keloid between 2010 and 2024 were analyzed. Patients received either conservative therapy or surgical excision with or without postoperative treatments, including intralesional triamcinolone (TAC), irradiation, silicone, compression, and laser therapy. Recurrence-free survival was assessed using Kaplan–Meier estimation, univariate analysis and multivariate Cox proportional hazards modeling. **Results:** Male sex, specific anatomical sites (ear and thorax), and ethnicity (Black/African, Asian, and Middle Eastern/Arab patients) showed significant associations with more recurrences. Univariate analyses indicated higher recurrence rates in patients treated with TAC or laser therapy, whereas irradiation, compression, and silicone showed no significant effect. Multi-component analysis revealed distinct patient clusters differing in recurrence burden and treatment patterns, and multivariate analysis showed that laser therapy remained associated with increased recurrence risk, whereas TAC, irradiation, silicone, and compression demonstrated modest protective trends. Combined use of the four latter modalities was associated with a non-significant trend to lower recurrence hazard (HR 0.75). **Conclusions:** This large European cohort highlights substantial demographic variability and heterogeneity in postoperative treatment strategies. Multimodal adjuvant therapy—particularly combinations of TAC, irradiation, silicone, and compression—may reduce recurrence risk, whereas laser-treated cases likely reflect confounding by indication.

## 1. Introduction

Keloids are chronic, fibroproliferative scars that extend beyond the original wound margins, cause pruritus, pain, and cosmetic disfigurement, and significantly reduce quality of life [1,2,3,4]. In contrast to hypertrophic scars, which occur stochastically, keloids have very high risk of recurrence after surgical removal. Thus, proper aftercare for prevention of keloid recurrence is crucial [5,6,7].

Current international and European guidelines recommend a multimodal approach to keloid management, with intralesional triamcinolone acetonide (TAC, Volon-A^®,^ Dermapharm Ag, Grünwald, Germany) typically administered every 4–6 weeks as first-line treatment, either alone or in combination with cryotherapy [3,5,6,7]. Additional treatment options include silicone gel or taping, compression, and laser-assisted drug delivery (particularly pulsed dye laser, PDL) are also recommended, especially for resistant or cosmetically sensitive lesions. For recurrent or extensive keloids, surgical excision followed by adjuvant radiotherapy remains the most effective strategy to reduce recurrence, with meta-analyses showing pooled recurrence rates around 10–20% compared with up to 90% after surgery alone [5,6,7,8]. Combination regimens, notably TAC + 5-fluorouracil (5-FU), have demonstrated superior efficacy over monotherapy in several randomized controlled trials and are increasingly integrated into stepwise treatment algorithms [9].

In addition to the therapies discussed above, a wide range of other treatment modalities for keloids have been investigated in recent years. First-line conservative options often include silicone gel or sheeting and pressure therapy to prevent progression and recurrence, frequently in combination with intralesional TCA [5,6,7,10] Adjuvant intralesional agents, including 5-fluorouracil, bleomycin, verapamil, botulinum toxin A, and hyaluronidase, have demonstrated variable efficacy and are increasingly used in multimodal regimens. Ablative and non-ablative laser therapies (e.g., pulsed dye laser and fractional CO_2_) may be beneficial either alone or in combination with injections to enhance drug delivery and improve scar texture and vascularity. Surgical excision remains an option for mature or refractory keloids, often combined with adjuvant radiation therapy of various modalities to reduce recurrence [5,6,7,10]. Additional approaches under investigation include topical immunomodulators such as imiquimod, systemic or local agents like pentoxifylline and interferons, as well as emerging biologic and cell-based therapies targeting fibroblast proliferation and growth factor pathways [11]. Photodynamic therapy (PDT) involves photosensitizing agents activated by light in the treatment landscape [12]. Despite this broad spectrum of therapies, no single modality has been universally accepted as curative, and multimodal, individualized approaches tailored to scar characteristics and patient factors are often necessary.

Keloid incidence varies across ethnic groups, with the highest prevalence consistently being reported among individuals of Black/African descent, where regional studies estimate rates as high as 4–16% [13,14], followed by Asian populations with reported prevalence around 1–1.5% in large population-based cohorts [15,16]. In contrast, keloids are relatively uncommon in people of European ancestry, with recent UK data indicating excessive scarring in roughly 0.4% of White, 1.1% of Asian, and 2.4% of Black/African participants [15]. Hardly any studies are available from Arab and Middle Eastern cohorts, but smaller regional series suggest a similar susceptibility pattern to that observed in Black/African and Asian groups [17]. Thus, due to the high occurrences, these ethnicities are well reflected in various studies, while there is limited data on keloids in White European populations, and we did not find comparable studies from Germany or Austria. Despite several small European studies investigating surgically treated keloids, most published cohorts are relatively small and heterogeneous, often restricted to single anatomical regions or specific adjuvant protocols such as postoperative radiotherapy [18,19].

Even though clear guidelines are available for clinical treatment algorithms [7,20], we found that, in our daily clinical work, these guidelines failed to provide sufficient recurrence prevention for many complex cases. Some treatment options, e.g., Deprodone tape [20], are not available in Europe. Moreover, we noticed that treatment approaches have changed over the years in our department, ranging from irradiation to laser to TAC only, but no clear consensus was found for the best therapeutical standard.

Therefore, we aimed to establish one of the largest Middle-European keloid cohorts to date, providing comprehensive clinical, demographic, and outcome data across a broad patient population and treatment spectrum.

## 2. Methods

This study involves patients treated in our department from 2010 to 2024. Data was extracted from the institutional keloid database, which has prospectively collected demographic and clinical information on patients treated either conservatively or surgically over more than 10 years. Each database entry corresponded to a single patient. For the present analysis, patients were categorized according to their primary treatment modality as either *conservative*, defined as the absence of any surgical procedure and usually involving intralesional triamcinolone injection, or *surgical*, defined as at least one documented operative excision of a keloid. A total of 319 records were screened and, after removal of empty or incomplete entries, 206 patients were included in the study population.

### 2.1. Ethics Statement

The Medical University of Vienna ethics committee (EK-Nr 2198/2024) approved the investigation of the retrospective patient data in this study.

### 2.2. Patient Cohort

Patients were eligible if they had been treated for at least one keloid at the Division of Plastic and Reconstructive Surgery, were 12 years of age or older at the time of treatment, and had received at least one form of follow-up therapy such as TAC injection, compression, silicone coverage, laser therapy, or adjuvant irradiation. Patients were excluded if they had undergone systemic chemotherapy or radiotherapy for malignant disease, if follow-up treatment had been performed entirely outside our department, or if their keloids originated from burn scars involving more than one percent of total body surface area, as such lesions frequently exhibit mixed hypertrophic and keloidal characteristics that preclude reliable classification.

Demographic and clinical parameters were extracted from electronic records of clinic visits and operative reports. The collected data included age at treatment, sex, and self-reported ethnicity (Caucasian, Black/African, Asian, Hispanic, or Turkish/Arab/Middle Eastern origin). Clinical variables comprised the anatomic location and body region of the keloid, the presumed etiology (injury or operation, burn, infection, or spontaneous onset), the number of keloids, the maximal keloid diameter in millimeters, and the type of resection, which was coded as complete, intralesional, or partial resection. Treatment-related parameters encompassed the number and dates of operations, pre- and postoperative TAC applications, postoperative laser treatment, compression or silicone therapy, and postoperative irradiation. Outcome variables included the presence of recurrence, the number of recurrences, and the time interval between the last surgical intervention and the first documented recurrence in months.

### 2.3. Definitions

#### 2.3.1. Keloid

In all patients with the diagnosis of “keloid” noted as ICD10 code L91.0, records were screened upon the definition of elevated, protruding scars extending beyond the original skin damage or as scars that were later defined as “keloid” in the histology report after resection.

#### 2.3.2. Recurrence

Recurrence was defined as newly detectable fibrous tissue growth exceeding 5 mm beyond the previous scar margin. This threshold reflects a pre-defined institutional standard rather than a guideline-based definition, as current national and international guidelines describe keloid recurrence descriptively without specifying quantitative cutoffs.

#### 2.3.3. Surgery

Surgery was performed either under general anesthesia or in local anesthesia, typically using Prilocain 1% (Xylanest^®^, AstraZeneca, Cambridge, UK) with additional adrenaline 1:10,000. Surgery was mostly performed as total marginal excision, and the entity of “keloid” was confirmed by histological report. Unfortunately, most surgical and histological reports did not fully comprise information whether the keloid was removed intralesionally or also into healthy tissue and, thus, this was not reported separately in the study. Dermal sutures were done using Ethilon (Ethicon, Raritan, NJ, USA), and stitches were removed between 7 days (surgery in the face) and 14 days after surgery. If longitudinal keloids were removed, Z-plasties were used, and in rare cases a skin transplant was needed for closure.

#### 2.3.4. Cortisone (Triamcinolone) Treatment

For postoperative intralesional cortisone treatment, 40 mg Triamcinolon (TAC) crystalline suspension (Volon-A^®^, Dermapharm Ag, Grünwald, Germany) was mixed 1:1 with Xylanest 1% in the same syringe and applied directly intracutaneously in the keloid. The exact amount differed according to the size of the keloid, but one dose or half a dose was used.

#### 2.3.5. Compression

For ear keloids, patients were recommended to purchase a compression ear clip according to their aesthetic preferences, making sure to cover the scar after keloid excision. For other body regions, patients were fitted with custom-made compression garments produced by an orthopedic technician. Sternal keloids were fitted with an additional compression pad. Patients were instructed to wear the compression clips or garments 24 h/day and only to remove them for showering or bathing for at least one year.

#### 2.3.6. Silicone

Patients were instructed to wear adequate silicone dressings covering the entire keloid or surgical scar after keloid removal (e.g., Mepiform^®^, Mölnlycke, Gotheburg, Sweden) for 24 h/day and to only remove for showering. Silicone dressings were typically worn for one year after excision.

#### 2.3.7. Laser

Before surgery, eligible patients were referred to the Dermatological department of the Medical University of Vienna for planning and scheduling laser therapy. Laser was performed postoperatively with dye laser with a 595 nm wavelength and a 0.5 ms pulse duration at the dermatological clinic in 5–6 sittings, each followed by application of Betemethason/Gentamicin ointment (Diprogenta^®^, Organon, Jersey City, NJ, USA).

#### 2.3.8. Irradiation

When planning surgery, selected patients were preoperatively assigned to the Department for Radiooncology at the Medical University of Vienna for scheduling irradiation. The first irradiation was done on the day of operation, and the following therapies in one-week intervals, with a total of 6 doses of 2.5 Gy ionizing irradiation each.

### 2.4. Statistical Analysis

All patient data was recorded in a Microsoft Excel table (Microsoft Office v16.0, Redmond, WA, USA). Further analysis, generation and statistical analysis of *t*-tests, Chi-square tests, frequency analyses, ANOVA and descriptive statistics were performed in GraphGad Prism (v8.0.1, Boston, MA, USA). Continuous variables were tested for normality and are presented as mean ± standard deviation (SD) or median with interquartile range [21] as appropriate. Comparisons between the conservative and surgical cohorts were performed using Student’s *t*-test for normally distributed continuous variables and the Mann–Whitney *U*-test for non-parametric data. Categorical variables were compared by means of the Chi-square test or Fisher’s exact test where applicable. A two-sided *p*-value of <0.05 was considered statistically significant. Multivariate statistical analyses were conducted using Python (version 3.11) with the *SciPy* and *pandas* libraries.

#### 2.4.1. Kaplan–Meier Curves

Recurrence-free survival curves were generated for each postoperative treatment modality—irradiation, postoperative TAC, compression therapy, silicone dressing, and laser treatment—comparing patients who received the treatment (≥1 application) with those who did not. Survival functions were estimated by the Kaplan–Meier method, and group differences were assessed using the log-rank (Mantel–Cox) test. All calculations were performed in Python (custom code) using standard survival-analysis algorithms equivalent to those implemented in the *lifelines* package [22].

#### 2.4.2. Multidimensional and Survival Analyses

To assess interdependencies between demographic variables and postoperative treatments, a multiple correspondence analysis (MCA) [23] was performed using all categorical and binary parameters, including ethnicity, body region, sex, and all treatment modalities (TAC, irradiation, compression, silicone, and laser). MCA reduces multidimensional categorical data into orthogonal dimensions that preserve the overall variance of associations, thereby allowing visualization of patients with similar treatment and clinical characteristics in a shared two-dimensional space. Cluster structure within the MCA embedding was subsequently examined in relation to recurrence frequency and treatment distribution.

To evaluate the independent impact of each variable on recurrence risk, a multivariate Cox proportional hazards regression model was fitted using the time from first to second operation (or to last follow-up in recurrence-free cases) as the survival time and recurrence (yes/no) as the event variable. The model included postoperative treatments, ethnicity, body region, and sex as covariates. Patients without recurrence were censored at the endpoint of follow-up. Hazard ratios (HR) and 95% confidence intervals (CI) were calculated, and the combined effect of the four beneficial adjuvant modalities (TAC, irradiation, silicone, and compression) was determined from the sum of their regression coefficients. Analyses were performed in Python using custom scripts based on *pandas*, *numpy*, and *lifelines* [22].

#### 2.4.3. Declaration of Generative AI and AI-Assisted Technologies

ChatGPT (version: GPT-5.1) was used for data visualization in this manuscript. The authors reviewed, edited, and take full responsibility for the final content.

## 3. Results

**Figure 1:**
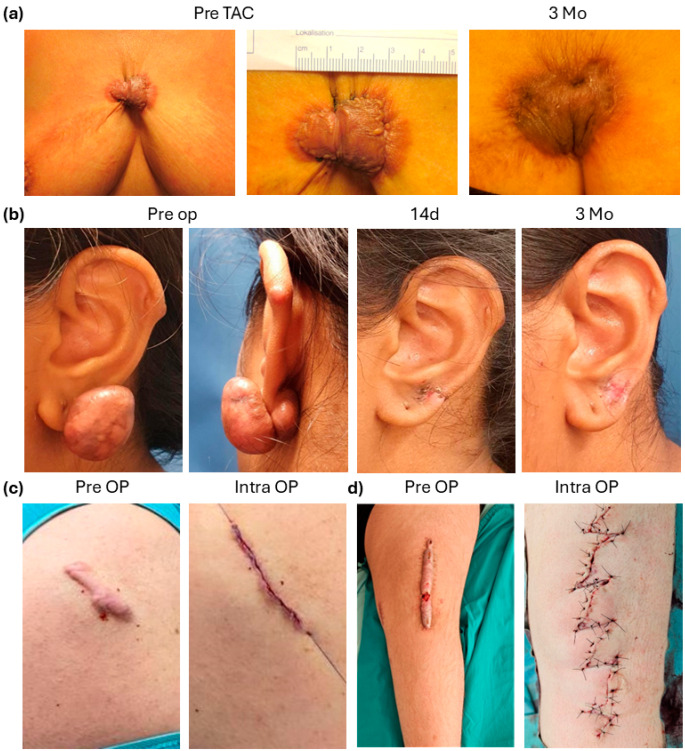
Conservative and surgical approaches to keloid treatment. (**a**) Female patient with painful sternal keloid before and 3 months after Volon-A injection, patient reported improvement of pain, tension and pruritus; (**b**) resection of large ear keloids with postoperative TAC and compression; (**c**) primarily resected shoulder keloid; (**d**) large keloid (20 cm) on the knee, excision and Z-Plasty performed.

In our cohort (n = 206), 49 patients did not receive any surgery, either because patients refused surgery, conservative treatment brought sufficient improvement of pain and pruritus (Figure 1a) or because sensible operative feasibility was deemed impossible by the surgeon. If operation was scheduled, keloids were operated by different methods, according to the anatomical location and the tension of the respective area; ear keloids were mostly resected at the base or with removal of a small wedge from the cartilage to allow tension-free closure (Figure 1b). If a keloid presented small enough in longitudinal orientation, primary excision and closure was done (Figure 1c). Only if very large, restricting keloids over joints were present, a Z-Plasty (Figure 1d) or, in rare cases, split-skin transplant was performed.

**Figure 2:**
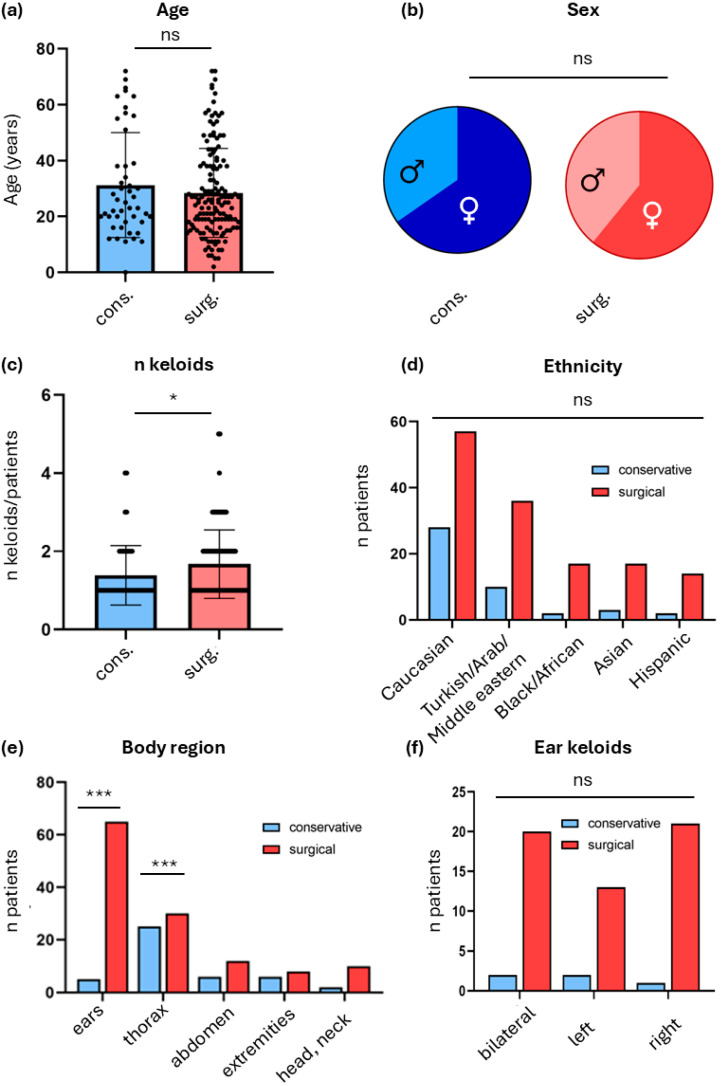
Demographic characteristics of surgically and conservatively treated patients. (**a**) Age distribution of all patients included in the study, mean age at treatment was 32 ± 15.4 years, no significant difference between conservatively and surgically treated patients (*p* = 0.31, *t*-test). (**b**) Sex distribution showing 62% female and 38% male patients (*p* = 0.61, Chi-Square test). (**c**) Number of keloids per patient. The median number was 2 (interquartile range 1–3) without significant difference in the number of keloids in conservatively vs. surgically treated patients. (**d**) Ethnic composition of the cohort, consisting predominantly of Caucasian (39%), Turkish/Arab or Middle Eastern (20%), Black/African (8%), Asian (7%), and Hispanic (7%), with no difference between groups, without significant difference in the numbers of conservative vs. surgical treatment between ethnic groups (*p* = 0.14, Chi-square test). (**e**) Distribution of keloids by body region. Ear, shoulder/thorax, and cervical/facial regions were most frequently affected. Ear and thoracic keloids were significantly more frequent among surgically treated patients (*p* < 0.001, Chi-square test). (**f**) Laterality of ear keloids. Left, right, and bilateral ear keloids were evenly distributed, and no lateral predominance or difference between frequency of surgical vs. conservative approach was detected (Chi-square, *p* = 0.33). ns = not significant, * *p* < 0.05, *** *p* < 0.001.

As conservatively treated patients cannot develop recurrence after surgery, they cannot readily be compared to surgical patients. Therefore, we first compared our cohort in demographic differences of non-operated vs. operated patients. In the total cohort, the mean age at treatment was 32.8 ± 16.6 years (range 12–83) with no significant difference between conservative and surgical cohorts (Figure 2a). The sex distribution tended to more female patients (56% female, 37% male) and was comparable across treatment modalities (Figure 2b). The number of keloids per patient ranged from one to fifteen, with a median of two (interquartile range 1–3) (Figure 2c). As this is a European study, ethnic composition was dominated by Caucasian patients (~39%) and Turkish/Arab or Middle Eastern (~20%), followed by Black/African (~8%), Asian (~8%), and Hispanic (~4%), without significant intergroup differences (Figure 2d). Analysis of body region revealed distinct anatomical predilections for keloid formation (Figure 2e). Lesions most frequently affected the ears and thoracic region, whereas abdominal, neck, and extremity keloids occurred less often. Surgical therapy was significantly more common for ear and thoracic keloids (*p* < 0.001), reflecting both functional and cosmetic treatment priorities. Analysis of laterality demonstrated comparable distributions of right, left, and bilateral ear keloids, with no significant difference between sides (*p* = 0.63) (Figure 2f).

**Figure 3:**
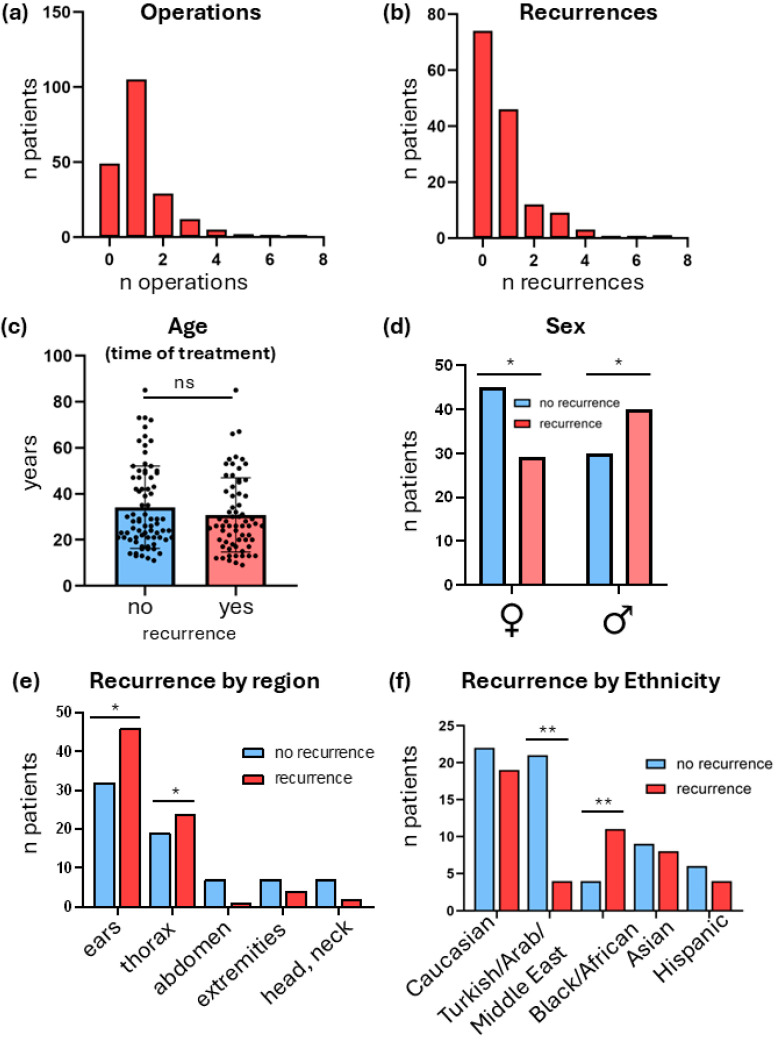
Clinical characteristics of surgically treated patient cohort. (**a**) Number of surgical operations per patient. (**b**) Distribution of the number of recurrences per patient. (**c**) Patient age at time of treatment in patients with or without recurrence (*p* = ns, *t*-test). (**d**) Sex distribution (*p* < 0.05, Chi-square test). (**e**) Recurrence frequency by body region. (χ2 = 11.03, df = 4, *p* = 0.026, Chi-square test). (**f**) Recurrence frequency by ethnicity (χ2 = 13.43, df = 4, *p* = 0.009, Chi-square test). ns = not significant; * *p* < 0.05; ** *p* < 0.01.

Next, we focused only on patients who had undergone at least one operation, as, naturally, only these patients are prone to recurrences. The number of operations per patient was predominantly one, with only a small subset requiring repeated excisions (Figure 3a) [24]. Likewise, the majority of patients experienced no or a single recurrence, whereas multiple relapses were uncommon (Figure 3b). Patient age at the time of treatment did not differ significantly between individuals with and without recurrence (Figure 3c). In contrast, male sex was associated with a higher recurrence rate than female sex (*p* < 0.05; Figure 3d). Analysis of anatomical distribution demonstrated significant variation in recurrence frequency between body regions (χ^2^ = 4.64, df = 4, *p* = 0.031), with the ear and thorax showing the highest recurrence rates, while abdominal, extremity, and head/neck sites recurred less frequently (Figure 3e). Recurrence rates also differed significantly among ethnic groups (χ^2^ = 13.43, df = 4, *p* = 0.009), with increased recurrence observed in Black/African patients and reduced recurrence among Turkish/Arab/Middle Eastern patients relative to other groups (Figure 3f). To further clarify the distribution of postoperative treatment modalities across ethnic groups, we calculated the relative frequency of each treatment within each ethnicity. Although numerical differences were observed (e.g., irradiation was most frequently applied in Black/African patients (60.0%), and silicone therapy was most common in Caucasian patients (40.0%), Chi-square analysis revealed no statistically significant differences in treatment allocation across ethnic groups (TAC χ^2^ = 6.52, *p* = 0.164; irradiation χ^2^ = 5.07, *p* = 0.280; compression χ^2^ = 5.31, *p* = 0.257; silicone χ^2^ = 3.84, *p* = 0.428; laser χ^2^ = 6.30, *p* = 0.178). Thus, postoperative treatment distribution did not significantly differ between ethnic groups in this cohort.

Next, we attempted to identify which commonly used postoperative treatments prevented recurrences most efficiently. In our department, readily available methods include TAC injection, postoperative irradiation, application of silicone dressings, compression garments or earclips, and laser treatment.

Recurrence-free survival was analyzed using Kaplan–Meier estimators. For each patient, the starting point (“time 0”) was defined as the date of the first keloid excision. The event of interest was a documented keloid recurrence. The time-to-event interval was determined either from the recorded *time until recurrence* or from the interval between the first and second operation or, if no second operation was performed, from the interval between the first operation and the patient’s last follow-up in the clinic (“endpoint”). Patients without recurrence were censored at their endpoint.

**Figure 4:**
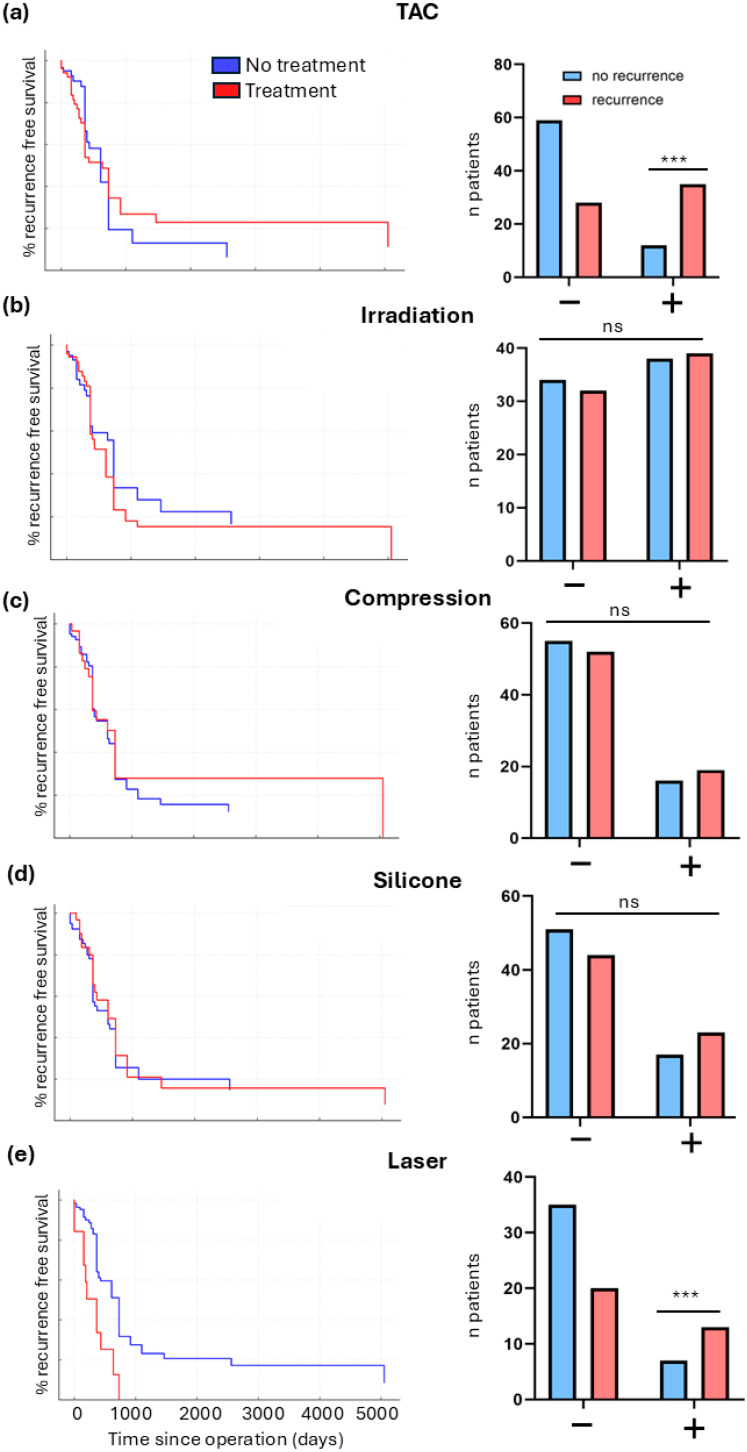
Impact of postoperative treatments on recurrence-free survival after keloid surgery. (**a**–**e**) Kaplan–Meier curves comparing recurrence-free survival of patients with (red) or without (blue) postoperative treatment, and corresponding bar graphs showing absolute numbers of patients with and without recurrence. (**a**) TAC, (**b**) irradiation, (**c**) compression, (**d**) silicone, and (**e**) laser (*p* < 0.001, Chi-square test). Laser-treated patients showed significantly higher recurrence rates and shorter recurrence-free survival compared with untreated patients. ns = not significant; *** *p* < 0.001.

Kaplan–Meier curves (Figure 4a–e) and Chi-square tests of treatment versus recurrence showed no significant association for postoperative irradiation, compression, or silicone (χ^2^ = 0.06, *p* = 0.79; χ^2^ = 0.34, *p* = 0.551; χ^2^ = 0.14, *p* = 0.235, respectively). Surprisingly, TAC-injected patients and laser-treated exhibited a significantly *higher* recurrence rate (TAC χ^2^ = 21.9, *p* < 0.001, laser χ^2^ = 4.881, *p* = 0.027) (Figure 4e).

**Figure 5:**
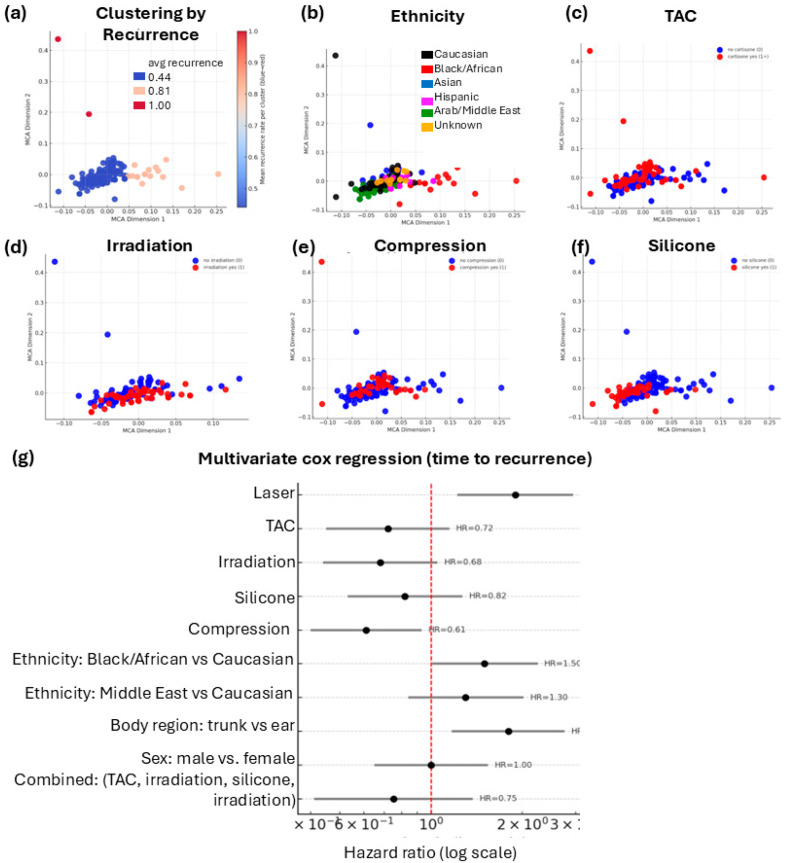
Multidimensional analysis of treatment combinations and recurrence risk. (**a**–**f**) Multiple correspondence analysis (MCA) visualizing the relationships between demographic variables, postoperative treatments, and recurrence. Each dot represents one patient positioned according to overall categorical similarity. (**a**) Clustering by recurrence frequency identified three patient clusters differing in recurrence rates. (**b**) Distribution by ethnicity and by postoperative treatments with (red) or without (blue): (**c**) TAC, (**d**) irradiation, (**e**) compression, and (**f**) silicone therapy. (**g**) Multivariate Cox regression analysis of time to recurrence including all demographic and treatment variables.

To account for the multiple confounding factors potentially influencing the efficacy of each individual postoperative treatment, we next performed a multiple correspondence analysis (MCA). This unsupervised multivariate approach allows visualization of relationships between categorical variables by projecting patients into a two-dimensional space defined by shared treatment and demographic characteristics. MCA revealed three major patient clusters that differed in their average number of recurrences (Figure 5a). These clusters showed distinct distributions in ethnic background (Figure 5b) and in the frequency of postoperative treatments applied, including TAC (Figure 5c), irradiation (Figure 5d), compression therapy (Figure 5e), and silicone sheeting (Figure 5f). In the subsequent multivariate Cox proportional hazards model (Figure 5g), and aligning with the univariate analysis, laser treatment was associated with an *increased* risk of recurrence (HR = 1.90). However, in contrast to univariate calculation, TAC, irradiation, silicone, and compression each showed slight protective trends with adjusted hazard ratios below 1.0. When the four adjuvant modalities were considered in combination, a lower hazard of recurrence was observed (HR = 0.75, 95% CI 0.41–1.38); however, this association did not reach statistical significance (Figure 5g).

## 4. Discussion

In the pertinent study, we present a comprehensive analysis of more than 200 patients with keloids in one of the first larger Middle European cohorts. Distinct ethnical distributions of were described before and were thus of particular interest [6,15,16,25]. In the Austrian general population, residents of Black/African and Arab/Middle Eastern origin together constitute well below 3% (Statistics Austria, Migration & Integration Report 2024) [26]. In contrast, these groups accounted for a substantially higher proportion of patients within our clinical cohort, indicating an overrepresentation among individuals presenting for specialist keloid care rather than reflecting population-level prevalence. This observation is consistent with prior reports describing a higher susceptibility to keloid formation in darker-skinned populations. Notably, our cohort also included a comparatively high proportion of patients of Arab/Middle Eastern origin, a group that remains underrepresented in the current keloid literature and for which epidemiological data are still limited [13,16].

A large U.S. retrospective cohort found that a numerically higher proportion of patients with keloids were non-White [6]. Moreover, a global review estimated that the average incidence of keloids may be 5–10% in Black/African populations versus <0.1% in lighter-skinned populations, highlighting substantial ethnic/racial differences [15,27]. Although our dataset does not permit direct calculation of keloid incidence in the Austrian general population, the disproportionately high representation of specific minority groups (Figure S2)—particularly patients of Black/African and Arab/Middle Eastern origin—relative to their population prevalence is consistent with previously described ethnic risk patterns for keloid formation. Ethnicity-related analyses in this study are descriptive and hypothesis-generating, as subgroup sizes do not allow reliable statistical comparisons between individual ethnic groups.

Our study found an anatomical site distribution dominated by ear (~38%) and thoracic/sternal keloids (~29%), followed by extremities and the abdomen, consistent with previous reports identifying these areas as keloid-prone [28]. We hypothesize that the clustering at these cartilage-adjacent regions (ear and sternum) may suggest local mechanical or biochemical factors influencing recurrence and growth, an interaction that was assumed before in vitro [29]. The in-depth analysis of keloids is limited by their relatively rare prevalence in the western population and thus naturally small cohorts and the complexity of their treatment. Our single-center approach offers the advantage of the exact documentation that treatments were documented per visit (e.g., silicone dressings and intralesional injections) and cross-department procedures (e.g., lasers performed in dermatology). This level of detail cannot be achieved in registry-only or ICD-screening datasets and enables nuanced analyses of what was done, when, and in what sequence.

However, this approach also comes with the limitations of the observational, non-randomized nature of our data, which leaves results prone to confounding by indication [30]. The higher recurrence rates observed in univariate analyses among patients receiving postoperative TAC or laser therapy must be interpreted in the context of the retrospective study design. In our clinical practice, these adjuvant treatments are preferentially assigned to patients with more severe disease, high-risk anatomical locations, or prior recurrences, resulting in a higher baseline risk of relapse. Consequently, increased recurrence in these groups does not imply treatment failure or a recurrence-promoting effect of TAC or laser therapy but rather reflects confounding by indication, a well-recognized limitation of observational studies. This interpretation is supported by our multivariate analyses, in which the univariate associations were attenuated after adjustment for relevant covariates. Together, these findings underscore the need for cautious interpretation of univariate treatment effects and for prospective studies with standardized treatment allocation.

Aside from this confounding by indication, our study is limited by the inconsistent treatment regimens over the years; from 2010~2013, laser therapies were performed more frequently in our department, while irradiation was used much later on (Figure S1). After 2020, TAC injections were preferred.

Moreover, prescriptions of compression and silicone dressings also differed between surgeons. During data acquisition, we also noticed that many patients had not adhered to their follow-up appointments. This might have occurred because some patients with recurrences were dissatisfied with their outcome and, thus, might not return for follow-up care and seek re-operation elsewhere.

An important aspect not considered in our data is comorbidities, which can significantly influence outcomes and add complexity to further analyses. A large British study found associations with hypertension in Black/African patients and vitamin D deficiency in Asian patients [2], as well as alopecia, rosacea, atopic dermatitis, and acne [6]. We aim to include comorbidity information in future analyses of our cohort.

We observed that postoperative treatments showed heterogeneous associations with recurrence. In the univariate analysis, TAC use demonstrated a significantly higher proportion of recurrences compared with no postoperative steroids, whereas irradiation, compression therapy, and silicone application did not show statistically significant differences. The most notable finding was the markedly higher recurrence rate in laser-treated patients, accompanied by visibly shorter recurrence-free survival. Because treatment allocation in our real-world clinical setting was not standardized, these results likely reflect confounding by indication, with clinicians preferentially offering laser (often together with TAC) to clinically more severe, recurrent, or high-risk lesions.

This pattern contrasts with the pertinent literature. Systematic reviews indicate that laser monotherapy shows variable efficacy, but laser combined with intralesional TAC or other agents can improve flattening and symptom control [10]. Nd:YAG-assisted TAC therapy has also shown benefit in postoperative management in prospective studies [31]. Likewise, evidence consistently demonstrates that excision alone carries very high recurrence risk, whereas excision followed by radiotherapy results in substantially lower recurrence rates across multiple series [10]. The German S2k guideline similarly recommends multimodal concepts, including excision plus adjuvant radiotherapy, intralesional TAC, silicone therapy, and pressure therapy, while noting that laser techniques should generally be applied as part of multimodal regimens rather than monotherapy [10]. Although the combined use of TAC, irradiation, silicone, and compression was associated with a trend to lower hazard of recurrence in our multivariate model, this effect did not reach statistical significance. The wide confidence interval likely reflects limited statistical power due to the relatively small number of patients receiving all four modalities simultaneously and the non-randomized allocation of treatments.

We assume that our divergence from the expected benefit of laser therapy therefore most likely reflects non-standardized protocols, variable laser types and parameters, and a selection of patients with expected high risk for recurrence, rather than a true detrimental effect of laser itself. These findings underscore the need for prospectively defined, parameter-controlled multimodal strategies, as well as careful risk stratification when integrating laser into postoperative keloid management.

Taken together, we hereby present a unique large European cohort with comprehensive multidimensional data as the basis for advancing a more accurate, clinically meaningful, and evidence-supported understanding of keloid disease.

From a clinical practice perspective, our findings support the consistent use of structured, multimodal treatment strategies for keloid management rather than reliance on single therapeutic modalities. No individual postoperative intervention achieved sufficient recurrence prevention when used alone, whereas combined approaches showed more favorable trends. This highlights the importance of routinely integrating established measures such as intralesional TAC, compression therapy, silicone dressings, and, where appropriate, adjuvant radiotherapy, tailored to patient and lesion characteristics. The higher recurrence rates observed in laser-treated patients further emphasize that laser therapies should be applied cautiously and preferably as part of standardized multimodal regimens. Finally, our results support the careful integration of emerging therapies, such as photodynamic therapy, into future treatment algorithms, particularly for refractory or high-risk cases.

## Data Availability

The full datasets presented in this article are not readily available because the data used in this study contains sensitive patient-specific data that may not be disclosed to external personnel. Requests to partially access the datasets should be directed to the corresponding author.

## Supplementary Materials

The following supporting information can be downloaded at: https://www.mdpi.com/article/10.3390/jcm15062150/s1, Figure S1: Relative frequency of treatments per ethnic group; Figure S2: comparison of the frequency of country of origin/ethnicities in the Austrian and Viennese population, compared to the study cohort; Figure S3: Temporal distribution of postoperative treatment modalities from 2010 onward.
