# Supplementary figures and images for: Clinical and Therapeutic Predictors of Keloid Recurrence: Outcomes in a European Cohort of 206 Patients
Source: J Clin Med. 2026 Mar 11;15(6):2150. doi: 10.3390/jcm15062150 (PMC13026513; doi:10.3390/jcm15062150)

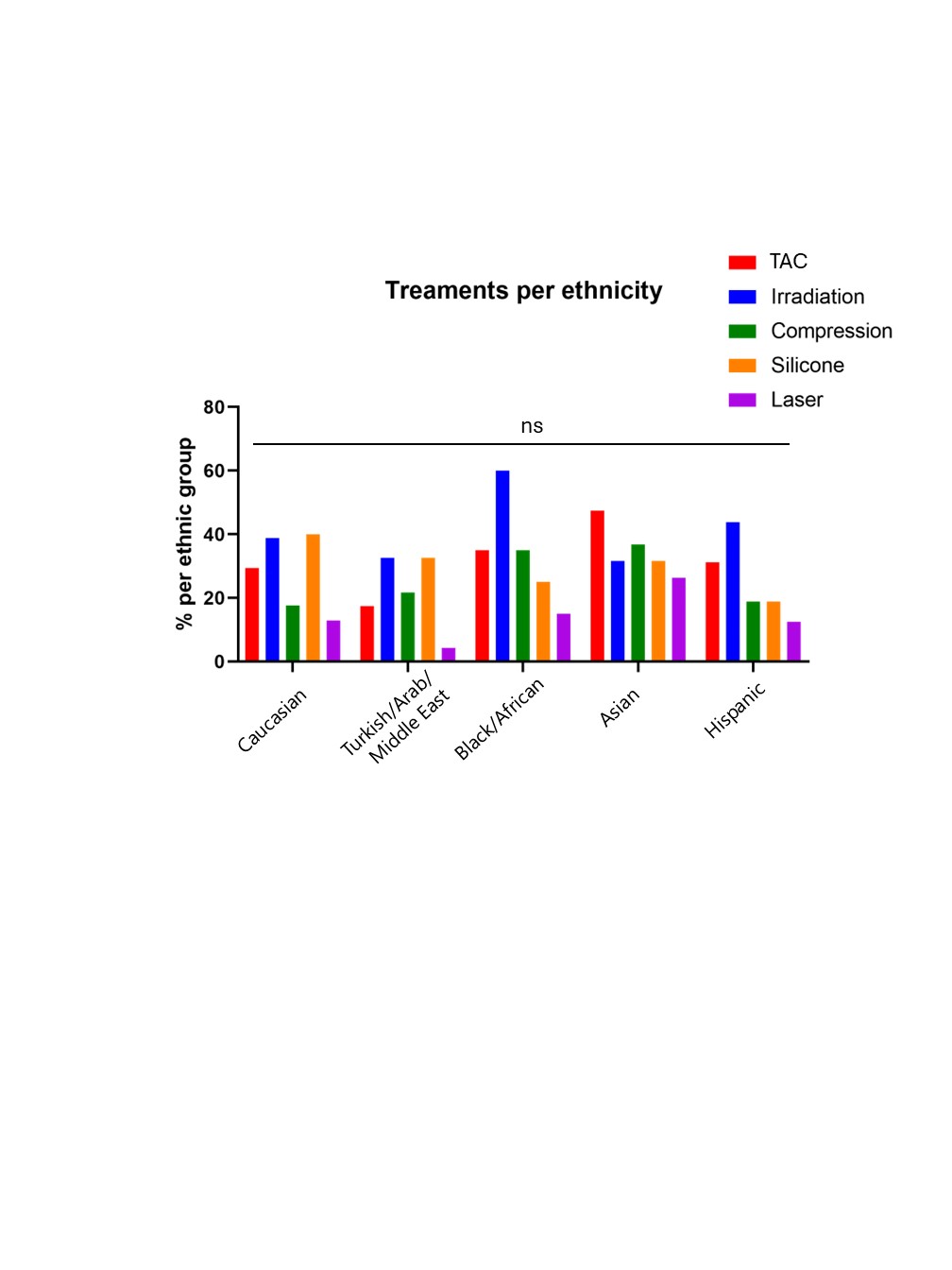

Supplement: Supplementary file 1 [file jcm-15-02150-s001.zip › Figure S1.jpg]

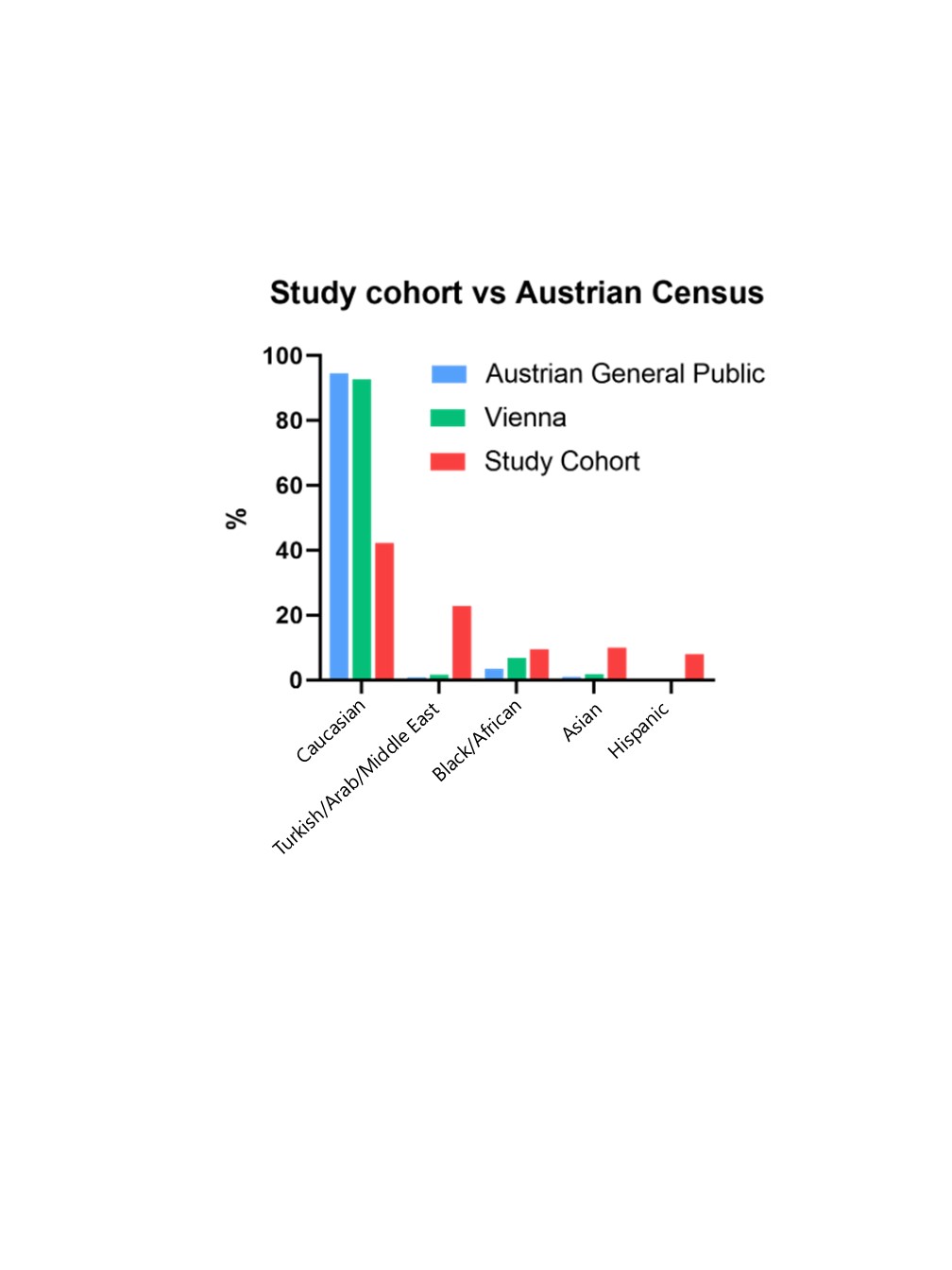

Supplement: Supplementary file 1 [file jcm-15-02150-s001.zip › Figure S2.jpg]

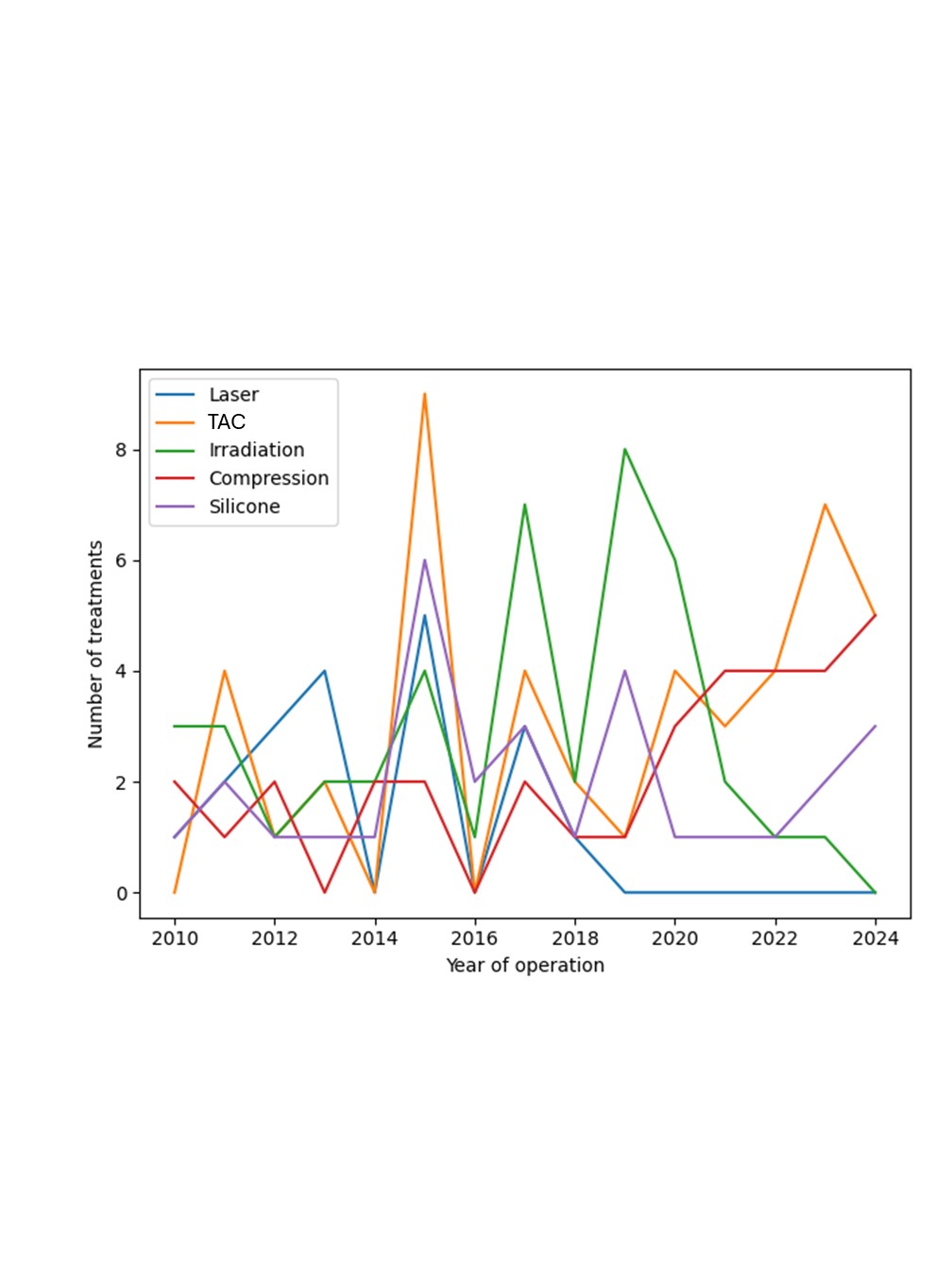

Supplement: Supplementary file 1 [file jcm-15-02150-s001.zip › Figure S3.jpg]
